# Colombia, an unknown genetic diversity in the era of Big Data

**DOI:** 10.1186/s12864-018-5194-8

**Published:** 2018-12-11

**Authors:** Alejandra Noreña – P, Andrea González Muñoz, Jeanneth Mosquera-Rendón, Kelly Botero, Marco A. Cristancho

**Affiliations:** 1 Bioinformatics Unit, Centro de Bioinformática y Biología Computacional de Colombia– BIOS, Manizales, Colombia; 2 Vicerrectoría de Investigaciones, Universidad de los Andes, Bogotá, Colombia

**Keywords:** Big data, Biodiversity, Latin America, Data mining, Molecular databases

## Abstract

**Background:**

Latin America harbors some of the most biodiverse countries in the world, including Colombia. Despite the increasing use of cutting-edge technologies in genomics and bioinformatics in several biological science fields around the world, the region has fallen behind in the inclusion of these approaches in biodiversity studies. In this study, we used data mining methods to search in four main public databases of genetic sequences such as: NCBI Nucleotide and BioProject, Pathosystems Resource Integration Center, and Barcode of Life Data Systems databases. We aimed to determine how much of the Colombian biodiversity is contained in genetic data stored in these public databases and how much of this information has been generated by national institutions. Additionally, we compared this data for Colombia with other countries of high biodiversity in Latin America, such as Brazil, Argentina, Costa Rica, Mexico, and Peru.

**Results:**

In Nucleotide, we found that 66.84% of total records for Colombia have been published at the national level, and this data represents less than 5% of the total number of species reported for the country. In BioProject, 70.46% of records were generated by national institutions and the great majority of them is represented by microorganisms. In BOLD Systems, 26% of records have been submitted by national institutions, representing 258 species for Colombia. This number of species reported for Colombia span approximately 0.46% of the total biodiversity reported for the country (56,343 species). Finally, in PATRIC database, 13.25% of the reported sequences were contributed by national institutions. Colombia has a better biodiversity representation in public databases in comparison to other Latin American countries, like Costa Rica and Peru. Mexico and Argentina have the highest representation of species at the national level, despite Brazil and Colombia, which actually hold the first and second places in biodiversity worldwide.

**Conclusions:**

Our findings show gaps in the representation of the Colombian biodiversity at the molecular and genetic levels in widely consulted public databases. National funding for high-throughput molecular research, NGS technologies costs, and access to genetic resources are limiting factors. This fact should be taken as an opportunity to foster the development of collaborative projects between research groups in the Latin American region to study the vast biodiversity of these countries using ‘omics’ technologies.

## Background

Colombia is one of the top countries that harbor the greatest diversity worldwide, due to high species richness for various taxonomic groups [1, 2]. This category is shared with other megadiverse countries, such as Brazil, Bolivia, China, Costa Rica, Ecuador, India, Indonesia, Mexico, Peru, South Africa, and Venezuela [3–6]. Currently, there are approximately 56,343 species reported for Colombia, including 7385 vertebrates, 20,647 invertebrates, 1637 lichens, 2160 algae, 30,736 plants, and 1637 fungi [7]. These numbers place Colombia as the second most megadiverse country worldwide without taking into account microbial species richness. Moreover, it holds first place in bird and orchid biodiversity; second in plants, amphibians, butterflies, and freshwater fish; third in palms and reptiles; and sixth in mammals [2, 4, 7, 8]. In order to maintain this great biodiversity, efforts for prioritizing and carrying out conservation strategies are necessary, based on biological, ecological, systematic, and, most recently, genetic knowledge of these species [2, 9, 10].

Information about genetic diversity is essential to optimize conservation strategies for biological resources and its uses, since molecular tools have allowed to identify genes implicated in a set of traits, including adaptive traits and polymorphisms that cause functional genetic variation [2]; gain insight into the functionality of an ecosystem as it has been shown in microbial communities regarding nutrient and energy flux [11]; assess the physiological condition of individual organisms as it has been shown in how endosymbiotic community shifts in corals depending on their health status [12]; study the philopatry of species, distribution and local adaptations by comparing neutral or conserved variations in the genome [13], as well as generate animal and plant breeding programs based on genetic markers [14].

Recent advances in high-throughput sequencing, molecular data generation, and bioinformatics have allowed to infer information about gene functionality, addressing with high specificity genetic variation at the individual level and how this variation represents the diversity at the phenotypic level, an adaptive trait and/or a marker of interest. In particular, what has significantly increased the achievements of molecular biology is bioinformatics, since this tool has enabled to read, interpret, and analyze huge amounts of data such as those generated from a high-throughput sequencing process in less time and with more accuracy. Some of the achievements of both molecular biology and bioinformatics include functional genomics, where is possible to study genes, proteins, and protein function, gene and protein expression in a cell under given conditions, 3D model generation in order to predict protein structure and function or pharmaceutical targets [15, 16], and presentation of molecular pathways in order to understand gene-disease interactions [17], among others.

Furthermore, a knowledge base of genetic resources is a useful approach to address food and nutritional security, crop improvement strategies and bioprospecting processes [18–21], as well as the possible processes that could be generated to benefit the sustainable development of a country [22]. In addition, efficient conservation and management plans can be implemented through the availability of comprehensive inventories of genetic resources, because the genetic variation of these resources can have a direct effect on the ability of species and populations to respond to environment changes through adaptation [10].

In Latin America, DNA sequence information generation and bioinformatics have advanced slowly compared to other regions of the world [23]. This is a relevant issue, given that molecular biology has reached the era of Big Data, through the ongoing development of high-throughput sequencing technologies that facilitate the massive generation of genetic data, leading to a widespread availability of sequence data in public databases [24–27]. Yet, the availability of public genetic data from Latin American countries is uncertain and little is known regarding the amount of molecular biodiversity data that is harbored in public databases.

In this study, we aimed to determine the amount of sequencing data of the Colombian biodiversity submitted by national institutions that is available in four main genetic sequence databases, including: Nucleotide and BioProject of the NCBI [28], Pathosystems Resource Integration Center (PATRIC) bacterial bioinformatics database [29], and Barcode of Life Data (BOLD) Systems [30]. We used a data mining approach, including data retrieval from the databases, data filtering, processing, and analysis. Furthermore, in order to obtain a broad and comparative view of the status of genetic diversity knowledge generation in Latin America, we compared this information for Colombia with other countries of high biodiversity in the region.

## Methods

We determined the level of representation of the Colombian biodiversity in the molecular data stored in public genetic sequence databases, and compared these findings with data for Latin American countries such as Argentina, Brazil, Costa Rica, Mexico, and Peru, as these countries are also harbor a high biodiversity.

The data mining approach included: 1. defining the databases to be searched; 2. defining search tools and criteria for data extraction from the databases; and 3. data processing and filtering.

### Databases

We searched in four public genetic sequence databases widely used by the scientific community, including: i) Genbank, specifically, the Nucleotide division. This database gathers all nucleic acid sequencing data from the DNA Databank of Japan (DDBJ), the European Molecular Biology Laboratory of the European Bioinformatics Institute (EMBL-EBI), and the National Center for Biotechnology Information (NCBI). (ii) BioProject, this database gathers all biological information and data related a to a single project and allows to retrieve information through related links that is sometimes difficult to find due to inconsistent annotations, multiple independent submissions, and/or because there are diverse data types that are usually stored in different databases iii) Barcode of Life Data (BOLD) Systems, that allows to obtain data of barcode sequences from the planet’s biodiversity; and iv) The Pathosystems Resource Integration Center (PATRIC) that represents a bacterial bioinformatics database.

### Search tools and criteria

Searches in the Nucleotide and BioProject databases were carried out using the Entrez Direct utility on the UNIX command line, which allowed data retrieval and formatting to generate customized downloads. The search criteria for the Nucleotide division was established as follows:

Esearch –db nucleotide –query “Name of the country” | Efetch –format gb.

Similarly, the search criteria for the BioProject division was:

Esearch db – bioproject –query “Name of the country” | Efetch –mode xml.

In BOLD Systems and PATRIC databases, searches were done by the term “Country” and the data was downloaded in TSV and Excel formats, respectively. For BOLD Systems, all barcode records were included, and for PATRIC, the record reports from the “Genomes” division were included.

### Data processing and filtering

Once the records for each country were retrieved from each of the databases, the data processing step involved counting the entries per country, using customized scripts written in *awk* programming language. We considered an entry to be published at the national level if the name of a national entity was mentioned in the respective search field in the databases. Afterwards, data filtering was also performed by customized scripts written in *awk* programming language. We classified the entries according to the following criteria: i) institutes that submitted data for each country and ii) organism or taxonomic group. Entries submitted by private or unknown collections were not taken into account, because of the lack of information about their origin. Table 1 shows the search fields that were taken into account in order to filter and count the entries per country and institution.

**Table 1:** Search fields for the respective databases in order to filter the entries produced at the national level

Databases	Search fields
Nucleotide	Journal
BioProject	<Submission <Organization <Name>
BOLD Systems	Institution_storing
PATRIC	Sequencing_center

## Results

The records shown here were retrieved from Genbank database release 219.0 of April 15 2017, containing 200,877,884 reported sequences, and the data from the other databases was obtained in June of 2017.

### Nucleotide

For the search term “Colombia”, 479,319 total records were found, with 320,420 sequences (66.84%) published in the country. Among these, 253,006 entries belong to the transcriptome assembly project titled “Transcriptome analysis of the Caribbean reef-building coral *Pseudodiploria strigosa* reveals a complex immune repertoire” [31]. Among the total entries, which represented 2462 species, the coral species *P. strigosa* has the greatest amount of records.

**Fig. 1:**
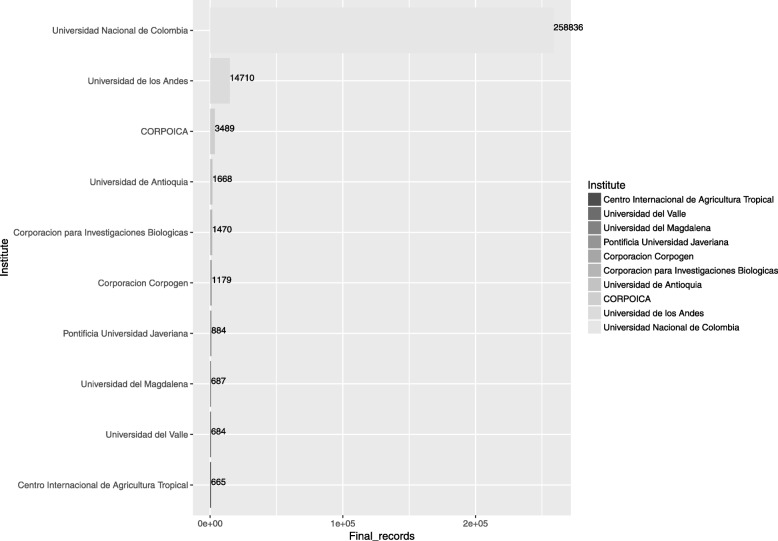
Main Colombian institutes that submit data to the Nucleotide (NCBI) database (Release 219.0 of April 15 of 2017). The values shown were compiled and analyzed in this study

We determined that 42 Colombian institutes have submitted information to this database, and among these, Universidad Nacional de Colombia stands out with 258,836 (80.78% of total records), followed by Universidad de los Andes, Corpoica, and Universidad de Antioquia (Fig. 1). Specifically, for the Eje Cafetero region (departments of Caldas, Risaralda and Quindío), which harbors a high number of universities and research centers, there are five institutes representing a total 1076 records. CENICAFE has the majority of entries (548), with 273 belonging to *Coffea* spp., Universidad Tecnológica de Pereira registers 419 records for six species (*Tabebuia rosea, Cordia alliodora, Heliconia orthotricha, Colletotrichum gloeosporoides, Rubus glaucus*, and *Alibertia patinoi*), while Universidad Católica de Manizales, Universidad de Caldas, and Universidad del Quindío show 67, 35, and 7 records, respectively.

As for other Latin American countries, Brazil has a total 2,098,579 records with 13.96% of these that have been published at the national level. Among the 67 national institutes identified, Universidad de Sao Paulo has provided most of the records (70.13%), followed by the Instituto Oswaldo Cruz, the Laboratorio Nacional de Computación Científica, and Universidade Federal do Rio Grande do Sul with 7611, 6658, and 6542 records, respectively. Nearly 3100 species were identified in the total records, of which the best-represented organisms were uncultured bacteria (108,611 records), *Acinetobacter baumanii* (22,319 records)*, Klebsiella pneumoniae* (21,559 records)*, Enterococcus faecalis* (15,136 records), and *Escherichia coli* (9941 records).

For Mexico, 157,797 records out of 1,349,367 were submitted by 63 national institutions, representing 11.69% of the total records. Universidad Nacional Autónoma de México (UNAM) has published the majority of records (47,302), while the Hospital de Pediatría CMN Siglo XXI, and the Instituto Politécnico Nacional occupy the second and third places with 22,204 and 8296 records, respectively. The best-represented organisms at the national level are *Helicobacter pylori* with 45,015 records, most of them provided by the Hospital de Pediatría, and uncultured bacteria with 32,789 records. Mexico has the highest number of represented species in this database with 7700 species, compared to other countries.

Argentina showed the highest number of records retrieved in the search (4,098,605), where 57 national institutes have submitted 4.72% of the records. The Instituto Nacional de Tecnología Agropecuaria has provided most of them, with a total of 91,488 records, where the fungi *Puccinia sorghi* and the tree species *Prosopis alba* are the best-represented species. Universidad de Buenos Aires follows with 27,178 entries, among which the bacteria *Inquilinus limosus* and Hepatitis C virus are the best-represented (2235 and 2208 records, respectively).

Meanwhile, Costa Rica has a total 362,192 records, with 6388 (1.76%) published by national institutes. Overall, eight institutes have submitted data for 705 species, led by the Instituto Nacional de Biodiversidad, publishing most of the records (3880). The best-represented organisms belong to uncultured bacteria, with 3455 records, followed by *Clostridioides difficile* with 313 records.

Finally, 1.01% of the total records for Peru (645,753) have been deposited by national institutes. We identified 33 national institutes that have submitted data, among which Universidad Nacional Mayor de San Marcos holds the majority of records (2555), followed by the Instituto Nacional de Salud (884), Universidad Nacional Agraria La Molina (456), Universidad Nacional de la Amazonia Peruana (378), and Universidad Peruana Cayetano Heredia (339). A total of 494 species were identified, and the best-represented organisms were influenza A virus with 3478 records, followed by *Pasteurella multocida, Vibrio parahaemolyticus, Bartonella bacilliformis*, and the immunodeficiency human virus with 1543, 666, 407, and 318 records, respectively.

Table 2 shows the percentages of mammal, bird, reptile, amphibian, and vascular plant species representation based on nationally submitted genetic data available for each country in the Nucleotide database compared to referenced species diversity values.

**Table 2:** Species richness values for six Latin American countries, classified by taxonomic group, and percentage of species representation at a national level for each country compared to the referenced values of species diversity shown. The percentage values shown were compiled and analyzed in this study. Data was obtained from Genbank (Nucleotide) release 219.0 of April 15, 2017

	Species richness value per taxonomic group					
Country	Mammals	Birds	Reptiles	Amphibians	Vascular Plants	References
Colombia	492	1921	606	803	51,220	[7, 51–53]
Brazil	701	1712	793	1042	56,215	[51, 52, 54, 55]
Mexico	564	1113	922	382	26,071	[51–55]
Argentina	386	1049	364	439	10,593	[51, 52, 56, 57]
Costa Rica	249	918	259	205	12,119	[51, 52, 54], [53, 58, 59]
Peru	441	1781	484	592	17,144	[51, 52, 54]
Percentage of species representation at the national level (Nucleotide database) compared to species richness per taxonomic group						
Colombia	9.35%	4.79%	2.48%	13.33%	1.13%	
Brazil	10.27%	1.58%	0.38%	7.10%	0.82%	
Mexico	30.32%	25.34%	12.36%	35.60%	5.53%	
Argentina	26.94%	5.53%	38.19%	44.87%	12.90%	
Costa Rica	1.61%	0.11%	2.32%	0%	1.50%	
Peru	1.13%	0.06%	1.03%	0.51%	0.27%	

### BioProject

**Fig. 2:**
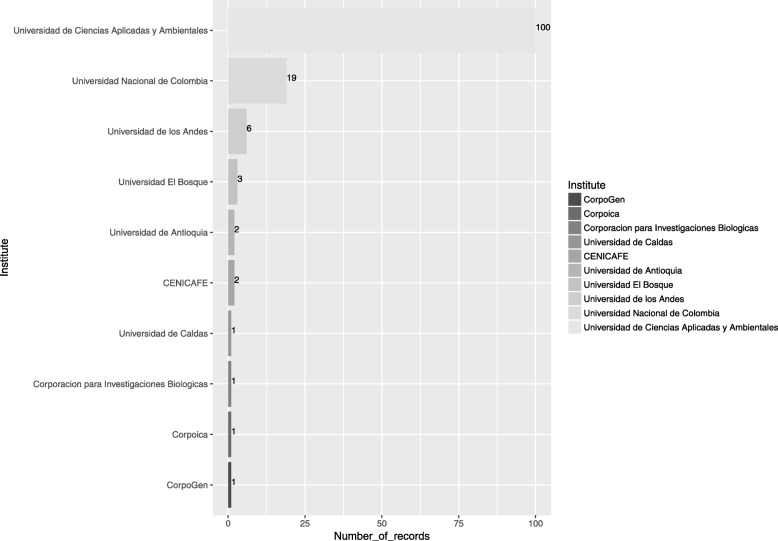
Colombian institutes that submit data to the BioProject (NCBI) database (Consulted June of 2017). The values shown were compiled and analyzed in this study

In general, Colombia, Brazil, and Argentina have more than 50% of records reported nationally, while Mexico, Peru, and Costa Rica show less than 22%. For the search term “Colombia”, 193 records were found, of which 136 (70.46%) have been reported by Colombian institutions. Universidad de Ciencias Aplicadas y Ambientales has provided the majority of records (Fig. 2), all of them for the bacteria *Helicobacter pylori*.

Regarding the Eje Cafetero region, there are only two institutes represented in this database, where CENICAFE has reported records for two species, two for the fungal species *Hemileia vastatrix* (BioProject accessions: PRJNA235221 and PRJNA188788 [32] and another for *Beauveria bassiana* Bb 9205 (BioProject accession: PRJNA165177 [33], while Universidad de Caldas has published data on the insect *Cosmopolites sordidus* (Bioproject accession: PRJNA291033).

For Brazil, of the total 558 records reported, 317 have been generated by national institutions, representing 56.8%. Of these, we were able to identify 90 belonging to the Empresa Brasileira de Pesquisa Agropecuária (Embrapa), Universidad de Sao Paulo, Universidad de Minas Gerais, and the Laboratorio Nacional de Computación Científica. Among these data, the best-represented organism groups are bacteria (*Escherichia*, *Klebsiella, Staphylococcus,* and *Salmonella*), followed by invertebrates.

In Mexico, 38 national institutes reported 153 (21.85%) out of 700 total records. Universidad Autónoma de México stands out with 77 records for the country, followed by the Laboratorio Nacional de Genómica para la Biodiversidad (LANGEBIO) of the Centro de Investigación y de Estudios Avanzados del Instituto Politécnico Nacional (CINVESTAV) with 11 records. Furthermore, among these data, the best-represented organisms are *Pseudomonas, Rhizobium*, and *Salmonella*, in addition to *Homo sapiens.*

For Argentina, there are 93 records published, of which national institutes have produced 53.76%. The Instituto de Agrobiotecnología Rosario (INDEAR) has submitted most of these (12), followed by the Consejo Nacional de Investigaciones Científicas y Técnicas (CONICET) with nine records. A total of 36 organisms were found, being *Trypanosoma cruzi* and *Lactobacillus mucosae* the best-represented with three records each.

Costa Rica shows the least amount of records (40) in the BioProject database, and only three have been published by national institutions. Universidad de Costa Rica provided two records for *Clostridioides difficile* (formally *Clostridium difficile* [34]) (BioProject accessions: PRJNA293889 and PRJNA264745 [35])*,* and the Instituto Nacional de Biodiversidad submitted one marine metagenome (BioProject accession: PRJNA19735). Interestingly, the Joint Genome Institute (JGI) (USA) has provided the majority of records (25%) available for Costa Rica, through data of the termite gut metagenome (BioProject accession: PRJNA19107 [36]).

In Peru, 15.95% of 94 total records has been generated at a national level. Seven institutes have submitted data to this database, namely Universidad Nacional Agraria La Molina, Instituto Nacional de Salud, Universidad Nacional Mayor de San Marcos, Laboratory FARVET, Universidad Cientifica del Peru, Universidad Cayetano Heredia, and the UCSD Archaeological field School in Peru. Among them, the Universidad Nacional Agraria La Molina and Instituto Nacional de Salud have provided the most records, with five and four, respectively, spanning the microorganisms *Bradyrhizobium, Aspergillus, Mycobacterium*, and *Bartonella*.

**Fig. 3:**
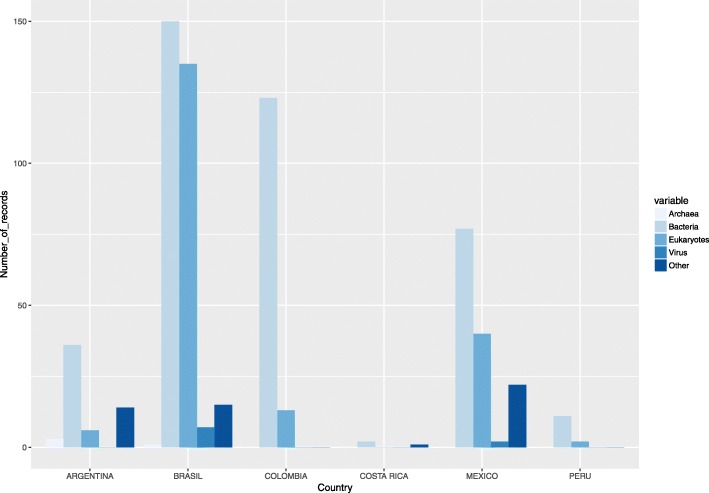
Number of records for each taxonomic supergroup in the BioProject (NCBI) database (Consulted June of 2017) for six Latin American countries surveyed. The values shown were compiled and analyzed in this study

In BioProject, it is noted that Bacteria holds the most records in all countries in terms of taxonomic supergroups representation (Archaea, Bacteria, Eukaryotes, Virus, Others), while the other supergroups are partially represented for the different countries. Brazil, however, shows data for the five supergroups (Fig. 3).

### BOLD systems

**Fig. 4:**
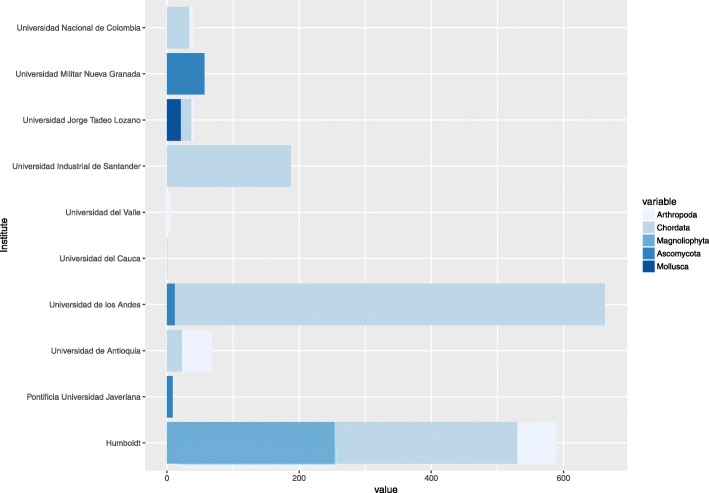
Colombian institutes that submit data to BOLD systems database and data representation of phyla per institute (Consulted June of 2017). The values shown were compiled and analyzed in this study

For this DNA barcode database, records from the public data portal as well the Barcode Index Number (BIN) were included. The search term “Colombia” retrieved a total 6457 entries, with 1673 (25.7%) submitted by national institutions. Universidad de los Andes and the Instituto de Investigación de Recursos Biológicos Alexander von Humboldt have provided the majority of records for the country, while there were no submissions found from institutes of the Eje Cafetero region. Moreover, 1261 species and five phyla are represented by the data, led by Chordata with 1189 records (Fig. 4).

In general, Phylum Arthropoda, with a total 194,499 records from all countries, has the highest representation, mainly from Argentina, Costa Rica, and Mexico. In addition, phylum Chordata comes in second, with 32,397 records, mostly represented from Argentina, Brazil, and Mexico.

We were not able to estimate species representation values for this database, since species-level information is lacking for many records in BOLD Systems database. However, in order to estimate the representation of these groups in terms of records (not species), we determined the overall number of barcode sequence records for mammals, birds, reptiles, amphibians, and vascular plants (Table 3). Table 3 shows that Costa Rica has the highest number of national records for insect barcode sequences than any of the other countries surveyed, and these records represent 92.2% of the total records for the country. Likewise, Argentina has a high representation of insect barcode records (79,298), which represents 88.1% of the national total records, and also shows the highest number of national records for bird barcode sequences. Strikingly, Colombia and Costa Rica have no records for mammals in this database. Costa Rica also has no records for birds, and Peru shows no records for reptiles. Overall, compared to the other countries, except for Peru, Colombia shows a low data availability in this database.

**Table 3:** Number of DNA barcode sequence records deposited at a national level in BOLD Systems database (Consulted June of 2017), classified by taxonomic group, for six Latin American countries surveyed. The values shown were compiled and analyzed in this study

Country	Total national records	Number of national records and percentage of total records per taxonomic group					
		Mammals	Birds	Reptiles	Amphibians	Vascular Plants	Insects
Colombia	1673	No records	281 (16.8%)	253 (15.1%)	655 (39.2%)	254 (15.2%)	127 (7.6%)
Brazil	19,330	546 (2.8%)	1150 (5.9%)	1 (0.000005%)	1662 (8.6%)	639 (3.3%)	5392 (28.0%)
Mexico	62,096	2458 (4.0%)	1105 (1.8%)	59 (0.0009%)	84(0.001%)	1908 (3.1%)	13,693 (22.1%)
Argentina	90,055	960 (1.1%)	2651 (3.0%)	581 (0.6%)	79 (0.0009%)	2 (0.00002%)	79,298 (88.1%)
Costa Rica	101,266	No records	No records	3 (0.00003%)	17 (0.0002%)	7563 (7.5%)	93,387 (92.2%)
Peru	3438	13 (3.8%)	1715 (49.9%)	No records	12(3.5%)	919 (26.7%)	312 (9.1%)

### PATRIC

**Fig. 5:**
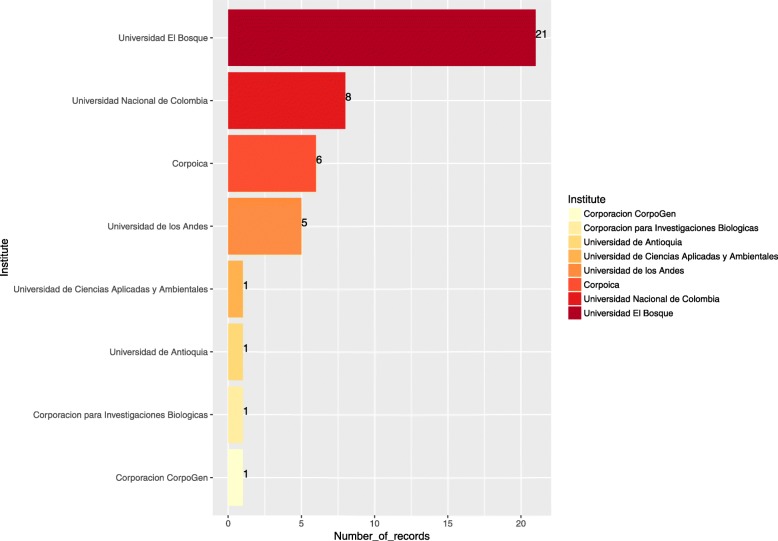
Colombian institutes that submit data to the PATRIC database (Consulted June of 2017). The values shown were compiled and analyzed in this study

A total 332 records were found for the search term “Colombia”, with 44 records published by national institutes, representing 13.25%. Universidad El Bosque has provided the majority of records at a national level (Fig. 5), all of them for different strains of *Staphylococcus aureus.*

Brazil showed the greatest proportion of records reported by national institutions, with a total 847 entries, where 401 (47.34%) were published by 39 national institutes. Universidad de Sao Paulo, with 128 records, has provided most of the records, followed by Universidad Federal de Para, Universidad Estatal de Campinas, and the Laboratorio Nacional de Computación Científica, each with 27 records. The best-represented organisms belong to the bacterial genera *Acinetobacter* and *Streptococcus*.

For Mexico, 165 out of 434 records (38.01%) correspond to data published by 18 national institutions, among which Universidad Nacional Autónoma de México (UNAM) has provided more than 65% (108 records). Data belonging to *Rhizobium* represent 30% of the total of records.

For Argentina, 487 records were obtained, and national institutions have contributed 9.65% of the data. The Instituto Nacional de Tecnología Agropecuaria, Universidad de Buenos Aires, and the Centro de Referencia para Lactobacilos (CERELA – CONICET) are the main institutes providing data for the country with 23 records altogether. In addition, organisms within the bacterial genera *Lactobacillus* and *Enterococcus* are the best represented.

Costa Rica and Peru reported 62 and 1737 records, respectively, and both show five records produced at the national level, representing 8.06% and 0.28%, respectively. In Costa Rica, only Universidad de Costa Rica has provided data corresponding to different strains of *Clostridium difficile*; while in Peru, Universidad Nacional Agraria la Molina and Universidad Nacional Mayor de San Marcos have reported three records for species belonging to *Bradyrhizobium*, *Acidithiobacillus ferrivorans* strain PQ33, and *Pasteurella multocida subsp. multocida* strain unmsm.

Furthermore, we considered genome status for the records and found that Whole Genome Shotgun projects dominate the records for all countries (83.9%), and the remaining percentage corresponds to Complete Genomes projects, except for Mexico, which also contains genomes in Assembly and Unfinished status.

## Discussion

This study involved the search for genetic sequence data available for Colombia and other Latin American countries in widely used public databases, in order to estimate biodiversity representation and national submission contribution in these databases. Genbank (Nucleotide division) contains the greatest number of records for the countries analyzed among all databases searched. However, for Colombia, approximately 78% of national records belong to a single transcriptomic study, also referenced in the Bioproject database [Accession: PRJEB6871 ID: 257057], and each of those records corresponds to a specific data set. Hence, the number of records in this database for Colombia may seem like a large number, but it does not encompass a large number of species. This is a clear example of the relevance of databases such as BioProject, since it can gather all information of a project in a single place allowing to easily access all of the data generated in the project without the need to search individually for sequences and avoid missing information. Finally, of the total biodiversity values reported in Biodiversity Information System (BIS) for Colombia, less than 5% of the Colombian species richness is represented at a molecular level in this database, even considering microbial genetic data [7].

Colombia’s history has likely played a role in limiting molecular data generation, due to diverse factors. One of them relates to the permits on access to genetic resources. In Colombia, genetic resources are property of the state, they are inalienable, imprescriptible and non-releasable, and access to them is regulated by the Andean decision 391 (*“Régimen Común sobre Acceso a los Recursos Genéticos*”), whereby whoever wishes to access them in the form of genes or derived products, according to the terms established in the decision 391, must request authorization of the state [37].

A study carried out by Quintero et al., in 2013 [14] regarding the status of access to genetic resources for Colombian research groups registered in Colciencias found that numerous research projects were being carried out without the necessary licenses, suggesting that a high percentage of national research was conducted illegally under the framework of the Colombian legislation. Some explanations for this behavior mention that existing mechanisms for biological research licensing and contracts for access to genetic resources are not time-effective, leading many scientists to give up on the process or not even try it. Also, the study mentioned that there was no relation between contract times and the time established to carry out the research, whereby a negative perception is generated both in national and international scientists for undertaking studies in the Colombian territory. In consequence, investigations that do not have the necessary permits cannot be published, such that the molecular information generated will remain publically unknown or known by a few in private databases.

Nowadays, the situation has not changed much, since according to another study carried by Silvestri in 2017 [38], there are still many complications regarding the access to genetic resources based on the legal framework, which lacks of regulation, seems to be inconclusive, in some cases non-specific, sometimes contradictory and not systematized. Besides, there is no specific legislation regarding the access to genetic resources in Afro and indigenous communities, so there is not a true participation of these communities in this subject matter. Given these issues, there is still “biopiracy” regarding studies made without the correct permissions [39]. There is also an issue with the timing and huge documentation and stages required to obtain a permit. Yet, luckily the law has helped to solve part of this issue, since law decrees 1375 and 1376 of 2013 establish that scientific investigation with non-commercial purposes which require biological species for molecular systematics, molecular ecology, evolution and biogeography do not need access to genetic resources permits (Decree 1375 of 2013, Decree 1376 of 2013).

Another possible limitation is related to the infrastructure and equipment needed to generate molecular data, as well as the associated costs of obtaining and using the equipment. In Colombia, there are several first, second, and third generation sequencers located in both public and private centers, including Universidad de los Andes, Instituto de Genética of the Universidad Nacional de Colombia, CorpoGen, International Center for Tropical Agriculture (CIAT), Corporación Colombiana de Investigación Agropecuaria (Corpoica), Universidad El Bosque, Universidad EAFIT, Corporación para Investigaciones Biológicas (CIB), among others. Nevertheless, the number of nationally available sequencers is low in terms of being competitive with North American and European countries and even in South American countries such as Brazil [40, 41]. This is in part due to the cost of obtaining sequencing equipment, ranging from 80,000 USD to over 125,000 USD [42], as well as a high cost of establishing and maintaining a sequencing facility such that it is competitive with sequencing prices abroad. This represents a great monetary value for countries such as Colombia, where high-throughput scientific research investment is often limited. Because of this, we usually resort to outsourcing services which are sometimes of bad quality because of inadequate sample handling, transport, and extended turnaround times [40].

In Colombia, interest in biotechnology, bioinformatics, and the “omics sciences” has risen in the last decade. However, support by the government is often one of the limiting factors for the development of related projects, due to scarce funding competed among a high number of national researchers. Since 2006, the gross domestic product (GDP) investment to the Science, Technology and Innovation sector in Colombia has increased from 0.401 to 0.736% in 2015. Nevertheless, in 2016, this investment decreased to 0.711% [43], demonstrating that the science and research sector has yet to become one of the national priorities. Government support for scientific research funding is managed mainly through the Administrative Department of Science, Technology and Innovation - Colciencias, which launches annual calls to fund Science, Technology and Innovation (STI) projects. However, due to the lack of financial resources dedicated to the STI sector in the country, which have always been less than 0.8% of the GDP [43], there are not many research projects that can benefit from this governmental aid. Also, a better classification and prioritization of high-impact projects is required, include projects based on omics technologies for data generation, which contribute high-value information to the country.

Fortunately, new scenarios are on the sight for Colombia. One of those being the post conflict scenario, an outcome of the peace agreement, where some areas that were once considered dangerous due to armed forces are now declared free zone, providing an opportunity to explore this places that have been off-limits for years. As an example, the country has recently started a major national initiative, Colombia BIO [18], which is exploring vast territories not reached before by scientists. Through several expeditions to these territories, the research community is already identifying new species at a great pace. These new species were unknown to science, because they inhabit places where armed groups dominated and did not give access to scientists. This endeavour is carried out by a large number of Colombian research centers and academia in collaboration with renowned international institutes, such as Kew Gardens in the UK. In addition, another program, namely BRIDGE Colombia [19], is also taking advantage of the new scenario proposing to explore broader geographic areas and aiming to increase our biodiversity figures, including genetic and taxonomic studies; all of this, in order to gain a better knowledge of our biodiversity’s potential that will benefit us both economically and socially.

Colombia has a better species representation in the Nucleotide NCBI database in comparison to other Latin American countries such as Costa Rica and Peru (Table 2). Furthermore, it is interesting that Mexico and Argentina have the highest representation of species at the national level, despite Brazil and Colombia, which actually hold the first and second places in biodiversity worldwide.

Due to lack of species-level information for numerous records in BOLD Systems database, we were not able to determine accurate species representation values nor compare these to the biodiversity values for each country (Table 3). On the other hand, regarding the amount of records submitted at the national level, Brazil leads the PATRIC database, while Colombia leads the Nucleotide and BioProject databases with a 65% higher national contribution than the other countries. Yet, for Colombia, most of the records belong to a single project for the species *Pseudodiploria strigosa.* This indicates that even though Colombian institutes may have produced a great amount of molecular sequencing data in relation to the other countries analyzed, these are not covering many more species.

During the last decade, some disciplines derived from molecular and genetic data have been gaining strength in Latin American countries, mainly because of the benefits they can bring commercially. Because of this, countries such as Mexico, Argentina, Brazil, Colombia, Chile, and Uruguay are leading in techniques such as genomics, biotechnology, and bioinformatics [44]. Biotechnology is the main sector that has considerably increased molecular data generation of biodiversity, due to its many applications. From 2005 to 2015, R&D investment in Latin America doubled, with Argentina, Brazil, and Mexico accounting for 91% of the total; consequently, these countries are regional leaders in biotechnology and genetic biodiversity data generation [45]. On the other hand, Costa Rica is a country where the use of high throughput technologies is not a priority, and its use has been mainly focused on investigating specific biological models and providing more data on these. The latter is also a consequence of low investment to the science sector, which limits this kind of studies [46]. A similar case is presented in Peru, where its economy is derived from exploiting natural resources, mainly mining, hydropower, gas, fisheries, forestry, metals and other minerals; thus, most funding goes to these fields. Meanwhile, in the science sector, the country has gone through a funding issue where the investment is exclusively targeted towards research and not to hire researchers. Consequently, there is a lack of appropriate personnel to develop projects involving, for instance, molecular biology, thus, resulting in less outcome of this kind of data [47].

Finally, it is hardly new that bacteria is the most commonly reported supergroup, since several of these microorganisms constitute an important cause of morbidity and mortality in humans and animals. Due to its importance, sequencing technologies have been very useful to understand much of its evolution, ecology, and pathogenesis, and have helped with the design of related therapeutic interventions. Furthermore, bacterial genomes are usually less than 5 MB, which facilitates its processing and assembly [48, 49].

## Conclusions

Overall, our findings show that the Colombian genetic biodiversity is under-represented in the consulted databases and that a great amount of records have been published by international centers and institutions. Among the factors that could be determining this scenario, we highlight the limited national funding for high-throughput molecular research, as well as lack of infrastructure for massive NGS data generation, which is restricted to a few sequencing equipments in public and private centers and institutions. Furthermore, the existing conflicts and obstacles in the granting of contracts of access to genetic resources can greatly limit the publication of genetic information generated in the country. Our work, therefore, manifests the need for greater government investment and cooperation to enable research and molecular data generation. In order to improve collaborative work it would be necessary to raise a greater interest and knowledge in the scientific and general community regarding the importance of describing our species, mostly at the molecular level. This will represent new resources for us as a society that can enable us to advance economically, for instance, to be used in biotechnology, or in pro of the conservation of our biodiversity which is also a priority for sustainable development. Collaborative work can be achieved by means of inter-institutional programs where different parties can contribute and gain benefit. Projects where a party can contribute with skilled personnel, another one with equipment, and another with regulatory framework that helps with obtaining and releasing data will foster collaboration in the country and benefit all parties associated, and progressively we could gain better academic and industrial relationships with developed countries.

Moreover, we highlight the importance of continuing scientific effort to publish data in public databases, which will promote that national information on genetic biodiversity can be freely accessible for research at a national and international level, contributing to place the country in a competitive position in scientific development worldwide.

Besides microorganisms, only a few of spider species, tropical parasites, and some insect vectors have been completely sequenced from the vast biodiversity of Latin America [23, 50]. This fact shows how the region has lagged behind in development of NGS and bioinformatics technologies to study the large number of species that are only present in this region of the world. To accomplish this, a greater number of collaborative efforts between Latin American and international scientists in regional and worldwide partnerships must be developed to promote research and publications in genomics fields and related science fields in the region.
